# Identification and catalytic properties of new epoxide hydrolases from the genomic data of soil bacteria

**DOI:** 10.1016/j.enzmictec.2020.109592

**Published:** 2020-09

**Authors:** Gorjan Stojanovski, Dragana Dobrijevic, Helen C. Hailes, John M. Ward

**Affiliations:** a Department of Biochemical Engineering, University College London, Bernard Katz, London WC1E 6BT, UK; b Department of Chemistry, University College London, 20 Gordon Street, London, WC1H 0AJ, UK

**Keywords:** Epoxide hydrolase, Limonene epoxide hydrolase, Genome mining, Biotransformation

## Abstract

Epoxide hydrolases (EHs) catalyse the conversion of epoxides into vicinal diols. These enzymes have extensive value in biocatalysis as they can generate enantiopure epoxides and diols which are important and versatile synthetic intermediates for the fine chemical and pharmaceutical industries. Despite these benefits, they have seen limited use in the bioindustry and novel EHs continue to be reported in the literature.

We identified twenty-nine putative EHs within the genomes of soil bacteria. Eight of these EHs were explored in terms of their activity. Two limonene epoxide hydrolases (LEHs) and one ⍺/β EH were active on a model compound styrene oxide and its ring-substituted derivatives, with low to good percentage conversions of 18–86%. Further exploration of the substrate scope with enantiopure (*R*)-styrene oxide and (*S*)-styrene oxide, showed different epoxide ring opening regioselectivities. Two enzymes, expressed from plasmids pQR1984 and pQR1990 de-symmetrised the *meso*-epoxide cyclohexene oxide, forming the (*R,R*)-diol with high enantioselectivity. Two LEHs, from plasmids pQR1980 and pQR1982 catalysed the hydrolysis of (+) and (−) limonene oxide, with diastereomeric preference for the (1*S*,2*S*,4*R*)- and (1*R*,2*R*,4*S*)-diol products, respectively. The enzyme from plasmid pQR1982 had a good substrate scope for a LEH, being active towards styrene oxide, its analogues, cyclohexene oxide and 1,2-epoxyhexane in addition to (±)-limonene oxide.

The enzymes from plasmids pQR1982 and pQR1984 had good substrate scopes and their enzymatic properties were characterised with respect to styrene oxide. They had comparable temperature optima and pQR1984 had 70% activity in the presence of 40% of the green solvent MeOH, a useful property for bio-industrial applications. Overall, this study has provided novel EHs with potential value in industrial biocatalysis.

## Highlights

• Five new active epoxide hydrolases were found.
• The limonene epoxide hydrolase from pQR1982 has a wide substrate scope.
• Enzymes from pQR1982 and pQR1984 had opposite epoxide ring-opening stereoselectivities.
• Enzymes from pQR1984 and pQR1990 de-symmetrised the *meso*-epoxide, cyclohexene oxide.
• The ⍺/β EH from pQR1984 had comparable activity in the presence of 5–30% MeOH.

## 1 Introduction

Enantiopure epoxides and diols are valuable chiral synthetic intermediates particularly in the fine chemical and pharmaceutical industries. Their preparation through traditional chemical means requires the use of expensive and toxic heavy metal catalysts with low catalytic efficiencies [1,2]. Epoxide hydrolases (EHs), enzymes which hydrolyse epoxides to vicinal diols, offer greener, sustainable and cost-effective access to these chiral synthons, providing high enantio- and regioselectivity without the requirement of expensive co-factors. These properties make them of considerable interest for bio-industrial applications.

EH enzymes are ubiquitous in nature, and have diverse biological functions including the detoxification of xenobiotics, regulation of signalling pathways and mediation of virulence [[3], [4], [5]]. Two distinct families of EHs have been identified. Most are members of the superfamily of ⍺/β hydrolases, containing a core domain of eight β-strands connected by ⍺-helices [6]. In contrast, limonene epoxide hydrolases (LEHs) are a minor family which are generally homodimeric and exhibit a unique fold composed of four ⍺-helices packed onto a six-stranded β-sheet which is highly curved [6,7]. The LEHs differ from the more common ⍺/β EH family as they have a unique active site, catalytic mechanism and little sequence similarity [7]. *Bona fide* LEHs are scantly reported in the literature, with few wild-type enzymes known including *Rhodococcus erythropolis* LEH (*Re*LEH), Rv2740 from *Mycobacterium tuberculosis* (*Mt*LEH) and two recently characterised LEHs from hot-spring metagenomes, Tomsk-LEH and CH55-LEH [[8], [9], [10]].

**Fig. 1:**
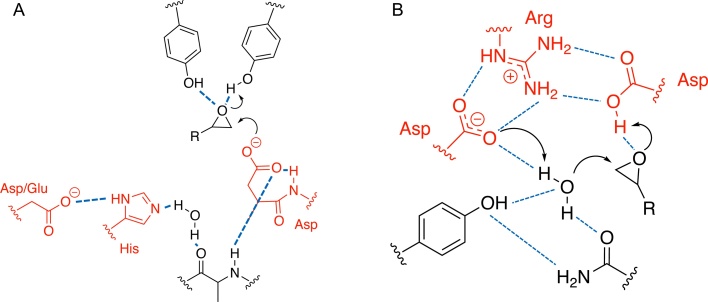
Diagrammatic representation of the two different modes of enzymatic epoxide hydrolysis. (A) 2-step mechanism of ⍺/β EHs proceeding via a substrate-enzyme intermediate. B) 1-step mechanism of LEHs. Residues of the catalytic triad of each enzyme type is shown red, H-bonds are shown in blue. Additional residues involved in stabilization of catalytic intermediates and orientation of substrates and water are shown in black. For references to colour in the figure legend, the reader is directed to the online version of this paper, doi:https://doi.org/10.1016/j.enzmictec.2020.109592.

The two EH enzyme families differ in their catalytic mechanism. ⍺/β EH enzymes have an Asp-His-Glu/Asp catalytic triad and utilise a two-step reaction mechanism (Fig. 1A). The epoxide ring is attacked by a nucleophilic aspartate residue forming a covalent substrate-enzyme intermediate [[11], [12], [13]]. A tyrosine residue donates a proton to the epoxide oxygen (Fig. 1A). The histidine residue of the catalytic triad acts as a general base activating a water molecule within the active site which attacks the alkyl-enzyme intermediate. The glutamate/aspartate residue of the triad interacts with the histidine residue as a charge-relay system, making activation of the active site water favourable [[11], [12], [13]]. The attack by water forms a tetrahedral intermediate and the negative charge which develops on the nucleophilic aspartate is stabilised by a single H-bonding interaction with the backbone nitrogen of the X residue in a conserved H-G-X-P motif (Fig. 1A) [14]. The tetrahedral intermediate formed dissociates to release the diol product.

In contrast, LEH enzymes catalyse the conversion of epoxide to diol via a concerted one-step mechanism (Fig. 1B). They contain an Asp-Arg-Asp catalytic triad and a tightly bound water molecule within the active site [7]. The water molecule directly attacks the epoxide, forming the diol without any intermediate enzyme-substrate complexes. One of the aspartate residues abstracts a proton from the water molecule, while the other donates a proton to the epoxide substrate [6,7]. The arginine residue stabilises the two catalytic aspartates via H-bonding interactions and regenerates their catalytically active protonated states [7,15].

EHs can furnish enantioenriched epoxides and 1,2-diols through a number of means. This can be through the kinetic resolution of racemic epoxides, the de-symmetrisation of *meso*-epoxides and enantio-convergent reactions [4,[16], [17], [18]]. These properties have potential value in industrial applications, and EHs have been reported in the synthesis of many important fine chemicals and pharmaceuticals [4]. For example they have been used to provide the (*S*)-enantiomer of ⍺-methylstyrene oxide, used to synthesise (*S*)-ibuprofen [19], the (*S*)-enantiomer of a side-chain precursor to (*R*)-Fridamycin E [20] and the (9*S*,10*R*)-9,10-epoxy-15-methylhexadecanoic acid used to synthesise the moth pheromone, (+)-disparlure [21]. In addition, EH enzyme kits are sold for use in commercial applications. For biotechnology applications, an enzyme with broad substrate specificity is desirable as it allows the biocatalyst to be used for a number of different biotransformations. Additionally, a tolerance towards organic solvents is essential as epoxide solubility can be a limiting factor. As such, biotransformations utilising EHs are commonly reported in the presence of a co-solvent, which can reach quite high reaction concentrations. For instance, EH reactions have been reported in the presence of 25–35% isooctane, 40% cyclohexane and even in a biphasic system where the epoxide forms its own phase [4,19,22,23].

Novel ⍺/β EHs and LEHs from nature have continued to be described in the literature. Using genome mining, van Loo and colleagues found six new ⍺/β EHs with broad substrate scopes [24]. Zhao and co-workers screened a library of EHs from environmental sources, finding several EHs capable of de-symmetrising *meso*-epoxides to provide enantioenriched 1,2-diols in high *e.e.* [17]. Kotik and colleagues found two enantioselective ⍺/β EHs from DNA isolated from a biofilter used to purify styrene-containing off-gas [25]. These two enzymes, Kau2 and Kau8, displayed opposite enantioselectivities, preferentially hydrolysing epoxides of (*S*)- or (*R*)-configuration, respectively [25].

More recently, novel metagenomic thermostable ⍺/β EHs and LEHs were recently described by Ferrandi and co-workers. The characterised LEHs had opposite enantiopreference for (±) limonene oxide to the archetypal *Re*LEH, slightly broader substrate scope and higher thermostability [10], while the ⍺/β EH, CH65-EH, is the most thermotolerant α/β EH described to date [26]. These examples highlight that nature continues to be a fruitful source for the discovery of novel EHs.

The aim of this study was to find new EHs with industrially useful activities by genome mining. We describe the identification of twenty-nine EHs from the genomic data of six soil bacteria isolates. Eleven EHs selected from two bacteria were successfully cloned and over-expressed in *Escherichia coli* and the substrate scope of eight of these enzymes was explored. Two of the enzymes had broad substrate scopes and were further characterised biochemically.

## 2 Materials and methods

### 2.1 General methods

All compounds were purchased from Sigma-Aldrich, Alfa Aesar or Acros Organics and used without further purification. Molecular biology reagents were purchased from New England Biolabs (NEB) and Thermo Fisher Scientific. DNA sequencing and oligonucleotides for PCR were performed and synthesised by Eurofins Genomics. Polyacrylamide gel electrophoresis (PAGE) gels were purchased from Bio-Rad. For routine cloning and expression, *E. coli* NovaBlue (Novagen) and BL21(DE3) (NEB) were used, respectively. Chiral HPLC analysis was performed on a HP Series 1100 HPLC with either a Chiralcel OB or Chiralcel OD column with a UV–vis detector. Chiral GC analysis was performed on an Agilent 7820A GC system fitted with a Supelco β-DEX 225 column (30 m x 250 μm x 0.25 μm) and a flame ionisation detector (F.I.D.) at 300 °C. Mobile phases and oven temperature ramps for the chiral analysis of diol products are detailed in Supplementary Tables S3 and S4.

### 2.2 Genome mining, multiple-sequence alignment and phylogenetic analysis of epoxide hydrolases

**Fig. 2:**
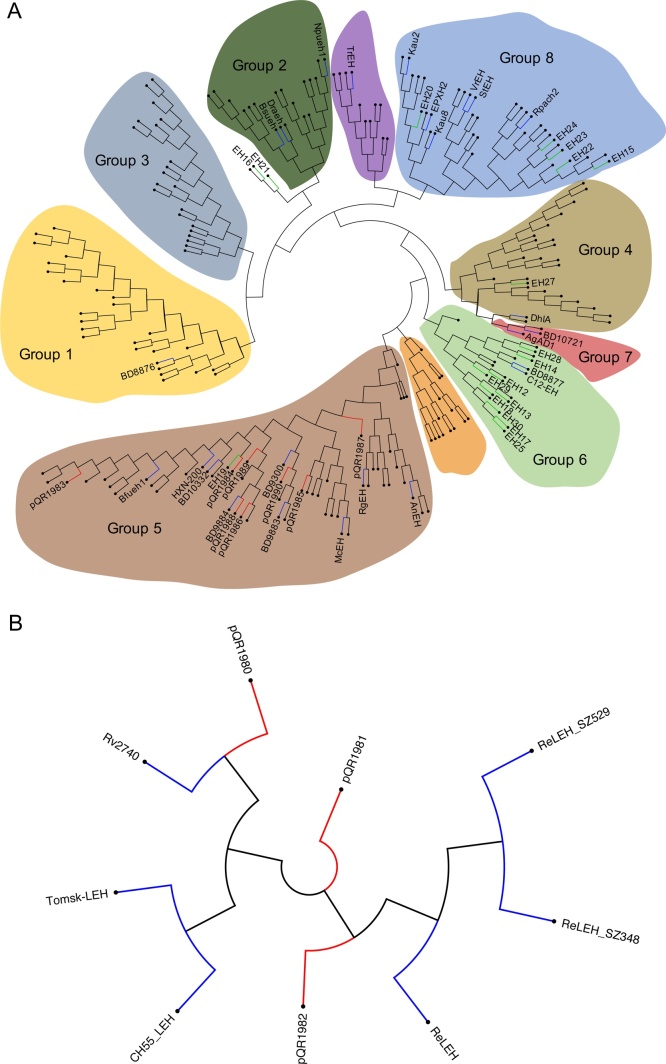
Phylogenetic analysis of ⍺/β EHs and LEHs. (A) Phylogenetic analysis of ⍺/β EHs. Separate groups are highlighted, with the group numbers indicating groups identified in van Loo et al. 2006. Red branches - EHs which were characterised further in this study, Green branches – other putative EHs found in this study, Blue branches – literature characterised EHs. The EHs BD8876, 8877, 9300, 9883, 9884, 10332, 10,721 are from [17]. (B) Phylogenetic analysis of LEHs. LEHs identified in our study are indicated with red branches, literature characterised LEHs with blue branches. For references to colour in the figure legend, the reader is directed to the online version of this paper, doi:https://doi.org/10.1016/j.enzmictec.2020.109592.

Putative EH sequences were mined for in the assembled genomes of six microorganisms using custom Python scripts. The microorganisms were the following: 1A1-3, a *Rhodococcus sp.* and 3A1-1, an *Aminobacter sp.* both soil isolates obtained from an enrichment culture; *Pseudomonas aeruginosa* M211, a strain isolated from a saline swamp in the Peruvian Amazon [27]; *Streptomyces violaceoruber* ISP5434 DSM40783; *Streptomyces griseolus* ATCC11796 and *Thermoactinomyces thalpophilus* THM1 [28]. The genes from each organism’s genome were compiled into a database containing 42,185 gene products. This database was queried for EH sequences by two methods; text mining of PROKKA or Prodigal annotations [29,30] that corresponded to EHs and searches for the following Pfam identifiers: epoxide N-terminal domain (EH-N) (Accession: PF06441) and LEH (Accession: PF07858). The thirty-three putative sequences found were used as queries to the National Center for Biotechnology Information (NCBI) BLASTp service to determine species origin and similarity to known EH sequences. These were passed into the ClustalOmega web service to generate a percentage identity matrix, which was visualised in Python 3 using the seaborn package. Twenty-six of the ⍺/β EH sequences were grouped with 185 ⍺/β EHs sequences from [24] and [20] sequences of literature reported ⍺/β EHs [11,13,17,18,24,25,[31], [32], [33], [34], [35]]. Similarly the three LEH sequences were grouped with six literature reported LEHs [7,9,10,36]. These were aligned using the MAFFT program with default settings in Jalview 2.10.5, and a phylogenetic tree was created using the maximum-likelihood method and 500 bootstrap repetitions in MEGA X. Fig. 2A and B was created using FigTree v1.4.4.

### 2.3 PCR amplification, cloning, expression and purification

Eleven putative EHs were amplified from the genomic DNA of two isolates, *Rhodococcus sp.* or *Aminobacter sp*. These were identified as pQR1980−1990 as per their designated plasmid code. PCR reactions were performed with the following conditions: 1 μM forward/reverse primers, 1 μL genomic DNA, 10% DMSO, 1.5 M betaine, 1x Phusion High Fidelity PCR Master Mix with GC buffer (Phusion MM buffer). Thermal cycling conditions for PCR were as follows: 98 °C/5 min, 30 cycles of (98 °C/10 s, 60−72 °C/30 s, 72 °C/45 s), 72 °C/7 min. Successful PCR reactions were confirmed by DNA gel electrophoresis. pQR1980−1989 PCR products were cut with *SapI* and ligated into a customised pET29a(+) vector (*D. Dobrijevic*, unpublished). The pQR1990 PCR product and the custom pET29a(+) vector were used in a circular polymerase extension cloning (CPEC) reaction following the procedure from [37] with the following conditions: 200 ng vector DNA, 3 x molar concentration insert DNA, 10 % DMSO, 1x Phusion MM buffer. PCR cycle conditions for CPEC: 98 °C/1 min, 18 x (98 °C/30 s, 52 °C/30 s, 72 °C/150 s), 72 °C/7 min. All constructs contained a C-terminal 6xHis-tag immediately following the last amino acid of the protein. For expression, cultures were grown to OD_600_ = 0.6−0.8 at 37 °C/180 rpm, induced with 0.1 mM IPTG and expressed at 25 °C/180 rpm shaking overnight. Successful expression was monitored by SDS-PAGE analysis. Cells were resuspended in lysis buffer (50 mM Na_2_HPO_4_ pH 7.4, 0.5 M NaCl, 20 mM imidazole), and were purified by immobilised metal ion affinity chromatography via the C-terminal 6x His-tag. Proteins were washed with lysis buffer containing a gradient of imidazole (20 mM, 50 mM, 100 mM, 200 mM) prior to elution with lysis buffer containing 500 mM imidazole. The final protein concentration was quantified by Bradford assay in triplicate. Proteins were precipitated with ammonium sulfate and stored at 4 °C. Prior to screening, enzymes were spun down 15,000 rpm/10 min and the precipitated protein resuspended and concentrated to 4 mg/mL in the assay buffer.

### 2.4 Enzyme activity screening

Activity assays were carried out with the following conditions: 50 mM sodium phosphate buffer (pH 7.4), 0.4 mg/mL purified enzyme and 10 mM epoxide substrate [dissolved in CH_3_CN or DMSO (10% final reaction concentration)] in a final volume of 250 μL. Reactions were incubated at 30 °C/300 rpm shaking for 20 h. The products were extracted 3 x with EtOAc and the organic layer dried with sodium sulfate. The solvent was removed under a N_2_/compressed air stream and the resulting residue was re-dissolved in 250 μL of EtOAc or iPrOH:Hexane (1:1) for chiral GC and chiral HPLC analysis, respectively. Yields were determined by calibration to an external product standard and assays were performed at least in duplicate.

### 2.5 Biochemical characterisation of the enzymes from pQR1982 and pQR1984 with respect to **1a**

Unless otherwise stated, all biochemical characterisation assays were performed at equivalent reaction concentrations as for enzyme screening and were terminated after 1 h incubation at 30 °C. Temperature optima screening was performed at 200 μL reaction volume and reactions were incubated at the following temperatures: 10, 21.3, 30.8, 40, 50.5, 60 °C. For time-course analysis, a 1.2 mL reaction was prepared, and 110 μL samples were taken at the following timepoints; 0, 5, 15, 30, 60, 120, 240, 480 and 1440 min post-reaction. Organic solvent tolerance assays were performed in a 200 μL reaction volume containing either MeOH, CH_3_CN or *tert*-butyl methyl ether (TBME) at the following organic solvent percentages: 5%, 10%, 20%, 30%, 40%, 50%. Substrate loading assays were performed as for enzyme screening, with the following modifications; final enzyme concentration was 0.1 mg/mL, **1a** was added yielding the following final concentrations: 1, 2, 5, 10, 20, 50 mM substrate and reactions were terminated after 30 min of incubation. All reactions were processed for chiral HPLC analysis using the same procedure as for enzyme activity screening. All assays were performed at least in duplicate.

### 2.6 Data analysis

HPLC/GC data was analysed in Python using custom scripts and the following packages; pandas, NumPy, matplotlib, seaborn, glob and Biopython. Integral values from HPLC/GC traces obtained from Agilent ChemStation software were averaged across replicates and converted to concentrations of product using a 5-point calibration curve from an external product standard. These concentration values were background corrected against a negative control without enzyme and converted to a % conversion value.

## 3 Results and discussion

### 3.1 Identification of new putative EHs and phylogenetic analysis

To find new EH sequences, the genomic data of six bacteria, four that had previously been isolated within our lab for their interesting metabolic and biocatalytic properties and two from bacterial culture collections, were searched. The six bacteria were strains of *Rhodococcus*, *Aminobacter*, *Pseudomonas sp.* M211, *Streptomyces violaceoruber* ISP5434, *Streptomyces griseolus* ATCC11796 and the thermophile *Thermoactinomyces thalpophilus* THM1 (Table S1). The *Rhodococcus* and *Aminobacter* strains were isolated from a soil enrichment from a location within a few meters of a motorway and these strains were believed to contain interesting enzymatic activity on organic soil contaminants. The gene sequences from these six bacteria were compiled into a database which contained 42,185 putative protein-coding sequences. By text-mining for 'epoxide hydrolase' annotations generated by Prodigal [29] and the Pfam identifiers for LEH and EH-N domains, three putative LEH and thirty ⍺/β EH sequences were identified. The eleven EH sequences (3 LEHs and 8 ⍺/β EHs) which were characterised further were labelled pQR1980−1990 per their designated plasmid code. The remaining twenty-two were labelled EH12−33. Subsequent analysis by BLASTp for similarity to known EH sequences, indicated that aside from the LEH, pQR1981, and the ⍺/β EH, pQR1989, which had 49–51% sequence identity to their highest hit, all other LEH and ⍺/β EH sequences had an average sequence identity of 99% to genes in sequence databases (Table S2). Eighteen sequences (3 LEHs and 15 ⍺/β EHs) alone were identified in the *Rhodococcus* strain and had high identity to genes from the recently sequenced *Rhodococcus sp.* ACS1 (Table S2) [38]. The two Streptomyces strains combined also contained 13 putative ⍺/β EH sequences. The number of EH sequences within these organisms may either reflect their larger genome sizes or the reported greater degree of secondary metabolism that occurs within these bacterial genera [39].

Initial multiple sequence alignment (MSA) of the putative EH sequences indicated the presence of three distinct groups: the LEHs, ⍺/β EHs lacking an N-terminal domain and ⍺/β EHs with an N-terminal domain (Figure S1). When the N-terminal domain sequence was removed, the group structure was maintained, indicating that differences between the two ⍺/β EH groups are also reflected in the C-terminal ⍺/β hydrolase domain, in agreement with previously reported findings [24]. Alignment of the ⍺/β EHs with the literature reported ⍺/β EHs from *Aspergillus niger* (*An*EH), *Solanum tuberosum* (*St*EH) and *Agrobacterium radiobacter* AD1 (AgAD1), and analysis of the conserved residues indicated high conservation of the catalytic triad (Figure S2A). All sequences contained a general histidine base, all but 4 (EH26, EH31, EH32 and EH33) had a catalytic aspartate, suggesting that these 4 might not be *bona fide* EHs and hence were excluded from subsequent analysis. The charge relay acid was less conserved, either being an aspartate or glutamate residue. It was also present in two possible positions, either aligning with D246 and D350 of AgAD1 and *An*EH, respectively or D217 of *St*EH. The presence of the charge relay acid in different positions has been observed by prior genomic analysis of ⍺/β EHs [24]. The tyrosine residue involved in the first step of epoxide hydrolysis was also highly conserved.

EHs of the ⍺/β hydrolase family have high sequence identity to fluoroacetate dehalogenases, the distinguishing feature being that EHs contain an aromatic residue at the X position in the H-G-X-P motif, and lack three conserved arginine residues following the catalytic aspartate in a DRXXRXXXR motif. They instead contain an aromatic residue immediately following the catalytic aspartate [24]. The MSA revealed that 26 of the ⍺/β EH sequences contained an aromatic residue in the H-G-X-P motif, this was either a tryptophan or phenylalanine. Non-aromatic residues at this position were also observed, either being valine or isoleucine. Additionally, none of the identified ⍺/β EH sequences contained a DRXXRXXXR motif, suggesting that the identified sequences were ⍺/β EHs and not fluoroacetate dehalogenases.

Similarly, an MSA for the three LEH sequences was conducted against the literature reported LEHs from *Rhodoccous erythropolis* (*Re*LEH), *Mycobacterium tuberculosis* (*Mt*LEH) and recently reported thermophilic LEHs, Tomsk-LEH and CH55-LEH (Figure S2B). This revealed complete conservation for the Asp-Arg-Asp catalytic triad and high conservation of the two residues involved in positioning the crystallographically observed water molecule which attacks the epoxide (Fig. 1B) [7]. This provided confidence that the putative LEH sequences were in fact LEHs.

Van Loo and colleagues [24] reported via genome analysis that there are eight phylogenetic groups of ⍺/β EHs, with only six of the groups at the time having at least one EH sequence which had been characterised in the literature. Hence, it was of interest to determine where our sequences fall in these eight families. Phylogenetic analysis was performed separately on all ⍺/β EH and LEH sequences identified in this study. For ⍺/β EHs, ten phylogenetic groups were identified (Fig. 2A). Eight of these groups were identified as equivalent to groups reported by van Loo and colleagues.

The ⍺/β EH sequences containing an N-terminal domain all grouped together along with the ⍺/β EHs from *Aspergillus niger*, *Rhodotorula glutinis*, *Mugil cephalus* and *Sphingomonas sp.* HXN 200. This group corresponded to group 5 reported by van Loo and colleagues, which contained animal, fungal and bacterial ⍺/β EHs and is the best characterised group to date, with the highest number of literature reported ⍺/β EHs. The members of this group are on average 100–150 amino acids longer than other ⍺/β EHs which is accounted for by the N-terminal domain [24]. The exact function of this domain is poorly understood. The N-terminal domain of mammalian soluble epoxide hydrolase (sEH) was shown to have phosphatase activity and contains a haloacid dehalogenase (HAD) N-terminal domain, yet in the ⍺/β EH from Aspergillus niger (*An*EH) the domain was suggested to be involved in enzyme dimerisation [13]. Sequence alignment of the N-terminal domains of our EH-N terminal domain containing proteins revealed greater identity with the EH N-terminal domain from *An*EH (35–45%) compared to the HAD domain of sEH (15–30%), suggesting that the N-terminal domain most likely lacks phosphatase activity, and may have similar function to that in *An*EH.

The ⍺/β EHs lacking an N-terminal domain were distributed across three of the other phylogenetic groups. EH15, 20 and 22–24 from the *Streptomyces sp.* grouped with the characterised ⍺/β EHs from *Solanum tuberosum* (StEH), *Vigna radiata* (VrEH), human soluble EH (EPXH2), Rpach2 [24] and the environmentally derived ⍺/β EHs, Kau2 and Kau8. This group corresponded to group 8 as reported by van Loo and colleagues which contains animal, plant and bacterial ⍺/β EHs. EH27 grouped with DhlA from *Xanthobacter autotrophicus,* which was in group 4 as reported by van Loo and co-workers and contains epoxide hydrolases and haloalkane dehalogenases. The presence of a phenylalanine in the H-G-X-P motif adds credence that EH27 is an ⍺/β EH as haloalkane dehalogenases tend to have more hydrophilic residues in this motif, like asparagine and glutamine. The other ⍺/β EH sequences grouped with the EH BD8877 and the EH from *Corynebacterium sp.* C12, indicating that they belong to group 6 reported by van Loo and colleagues which contained fluoroacetate dehalogenases. However, as already stated none of the ⍺/β EH sequences identified within this study contained the motifs typical of the fluoroacetate dehalogenases, instead containing characteristic ⍺/β EH sequence motifs. EH16 and EH21 appeared to form their own fringe group outside the main clusters observed (Fig. 2A).

Despite grouping with characterised ⍺/β EHs and the high conservation of the catalytic residues, overall sequence identity to literature characterised ⍺/β EHs was low, ranging from 15−50%. EH12 was an outlier in this regard as it was essentially identical to the *Pseudomonas aeruginosa* PAO1 CFTR inhibitory factor (Cif) bar one point mutation [40].

The LEH phylogenetic analysis suggested that pQR1980 was most similar to Rv2740 (*Mt*LEH), pQR1982 was most similar to *Re*LEH and pQR1981 was more distantly related (Fig. 2B). However, overall sequence identities were low. The LEH, pQR1980 has ∼ 45% sequence identity to *Mt*LEH and between 25–30% sequence identity to the other LEH sequences. The enzyme from pQR1982 had around 25–40% sequence identity while the enzyme from pQR1981 had around 25–30% identity to literature reported LEHs. Aside from EH12, to the best of our knowledge none of the EH sequences identified in this study have been previously reported.

### 3.2 Heterologous expression and purification of selected putative EHs

Our interest in exploring the bioindustrial applicability of new EHs led us to investigate eight of the EH-N terminal domain containing ⍺/β EHs (from pQR1983−1990) further, as by phylogeny they grouped with EHs which have already been successfully applied in biotransformations [17,[33], [34], [35],^41^]. The three LEH sequences (from pQR1980−1982) were also explored further as they are from a minor class of EH enzymes in which wild type enzymes have been scarcely reported in the current literature [[8], [9], [10]]. The EH sequences were cloned with a C-terminal 6x His-tag, over-expressed in *E. coli* BL21(DE3) and purified by Ni^2+^-NTA chromatography (Figure S3, S4). The enzymes from pQR1987 and pQR1988 despite having high levels of expression were highly insoluble while the enzyme from pQR1985 was expressed at low levels and wasn’t obtained in sufficient quantities for further characterisation. These enzymes were not explored further.

### 3.3 Exploration of the substrate scope of eight putative EHs

**Fig. 3:**
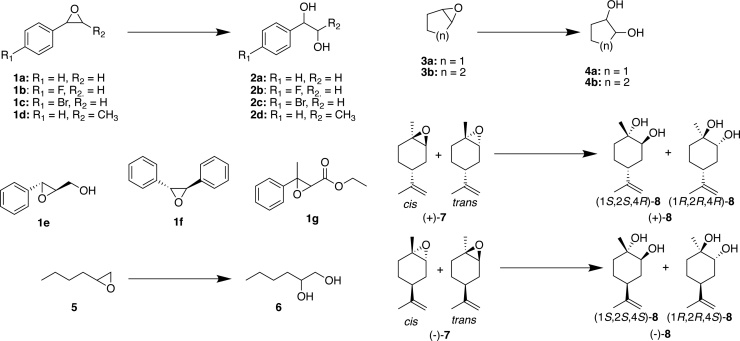
Panel of epoxides screened against the identified EHs. **1e**-**1 g** reactions were performed as for all styrene oxide derivatives, see Materials and Methods for details. **1c** and **3a-b** reactions used DMSO as a co-solvent.

Activity screening using a panel of 8 EHs and 15 epoxides [**1a**-**g**, **3a**-**b**, **5**, (±)-**7**] (Fig. 3) which produced diols [**2a**-**g**, **4a**-**b**, **6**, (±)-**8**] and contained a range of aromatic, aliphatic, cyclic, bulky and *meso*-epoxides indicated that the enzymes from pQR1980, pQR1982, pQR1984, pQR1986 and pQR1990 were active EHs (Table 1). The LEHs from pQR1980, pQR1982 and the ⍺/β EH from pQR1984 displayed moderate % conversions after 20 h reaction at 30 °C towards several of the epoxides tested, while the ⍺/β EHs from pQR1986 and pQR1990 had narrower substrate specificity and poor conversions (Table 1).

**Table 1:** Substrate scope and % e.e. of epoxide hydrolases towards [**1a-d**, **3b**, **5**, (±)-**7**].

**Enzymes**^a^										
	pQR1980		pQR1982		pQR1984		pQR1986		pQR1990	
Substrates	Percentage yield (%)^b^	% e.e. of diol (abs. config.)^c^	Percentage yield (%)^b^	% e.e. of diol (abs. config.)^c^	Percentage yield (%)^b^	% e.e. of diol (abs. config.)^c^	Percentage yield (%)^b^	% e.e. of diol (abs. config.)^c^	Percentage yield (%)^b^	% e.e. of diol (abs. config.)^c^
**1a**	29	6	66	2	65	13 (*S*)	3	0	8	20 (*R*)
(*R*)-**1a**	34	89 (*S*)	57	98 (*S*)	61	71 (*R*)	5	71 (S)	6	56 (*S*)
(*S*)-**1a**	22	87 (*R*)	57	96 (*R*)	86	94 (*S*)	5	72 (*R*)	8	77 (*R*)
**1b**	31	22 (*R*)	51	4	48	33 (*S*)	10	2	11	1
**1c**^d^	18	–	46	–	28	–	23	–	18	–
(1*R*,2*R*)**-1d**^e^	19	99	21	99	2	97	3	97	2	99
(1*S*,2*S*)-**1d**^e^	20	78	3	6	20	69	2	59	2	58
**3b**	11	22 (1*R*,2*R*)	46	17 (1*S*,2*S*)	22	84 (1*R*,2*R*)	1	14 (1*R*,2*R*)	26	88 (1*R*,2*R*)
**5**^d^	4	–	29	–	54	–	1	–	4	–
(+)-**7**	65	98 (1*S*,2*S*,4*R*)	Quant.^f^	86 (1*S*,2*S*,4*R*)	–	–	–	–	–	–
(-)-**7**	99	98 (1*R*,2*R*,4*S*)	87	99 (1*R*,2*R*,4*S*)	–	–	–	–	–	–

a Plasmid codes indicate the enzymes expressed from these plasmids.

b yields are for the diol products [**2a-d**, **4b**, **6**, (±)-**8**]. All yields are corrected for background chemical hydrolysis. “- “= no activity above background observed. All assays were conducted either in triplicate or duplicate and standard deviations were < ± 7%.

c Absolute configurations of (*R*)-**2a** and (*S*)-**2a** were determined by chiral HPLC of authentic standards. Absolute configuration of **2b, 4b** and (±)-**8** products were determined by comparison with literature reports [36,44,45].

d % e.e. of the diol product was not determined.

e Absolute configuration (abs. config.) of the diol product was not determined.

f Quantitative conversion.

The remaining EHs (from pQR1981, pQR1983, pQR1989) displayed little to no activity on the epoxides tested, suggesting that either these enzymes are inactive or that the substrates tested are not hydrolysed by these particular enzymes. The overall low levels of activity is not unprecedented, as all the LEH/α/β EH enzymes tested were isolated from a single *Rhodococcus* strain. It is possible that, one or two of the EH enzymes identified are involved in the detoxification of harmful genotoxic epoxides and hence have broader substrate specificity, whilst the rest have evolved to have more specialised roles and hence have narrower substrate scopes. The performance of the most productive enzymes is discussed further below.

**Fig. 4:**
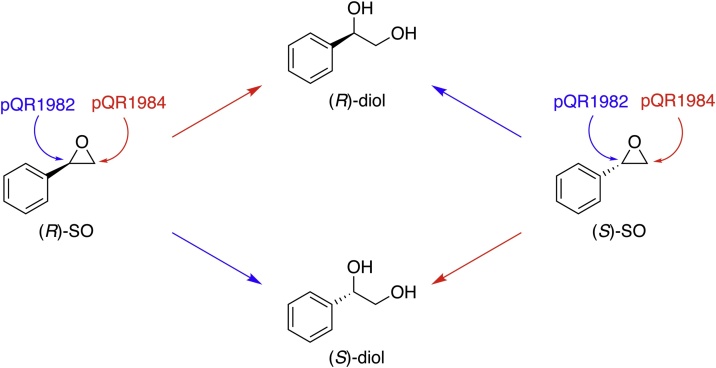
The LEH from pQR1982 and ⍺/β EH pQR1984 have opposite regioselectivities. The enzyme from pQR1982 produces the (*S*)-diol from (*R*)-styrene oxide, while the enzyme from pQR1984 produces the (*R*)-diol. The enzyme from pQR1982 produces the (*R*)-diol from (*S*)-styrene oxide, while the enzyme from pQR1984 produces the (*S*)-diol. SO = styrene oxide.

Styrene oxide (*rac*-**1a**) was accepted by all active EHs, with the enzymes from pQR1980, 1982 and pQR1984 having moderate conversions of 29–66% (Table 1). The enzymes from pQR1986 and pQR1990 had poorer conversions (Table 1). For racemic **1a**, all enzymes had low enantioselectivity (Table 1). Interestingly, when enantiopure (*R*)-**1a** and (*S*)-**1a** were used as substrates a difference in regioselectivity was observed (Table 1, Fig. 4, S5). The enzymes expressed from pQR1980, pQR1982, pQR1986 and pQR1990 all yielded the (*S*)-diol from (*R*)-**1a** and the (*R*)-diol from (*S*)-**1a**, indicating an inversion of configuration at the benzylic carbon (Table 1). The LEH from pQR1982 produced both diol products with excellent stereoselectivities (Table 1). However, the ⍺/β EH from pQR1984 had the opposite regioselectivity, yielding (*S*)-**2a** from (*S*)-**1a** and (*R*)-**2a** from (*R*)-**1a** in 94% *e.e.* and 71% *e.e.,* respectively (Table 1). This suggested that despite little kinetic resolution of racemic **1a**, the enzyme from pQR1984 appears to favour opening the epoxide ring on the terminal carbon similar to that of *An*EH [41], while all other EHs active towards **1a**, open up the epoxide ring at the benzylic carbon. The enzyme from pQR1984 was also less regioselective for (*R*)-**1a**, as some (*S*)-**2a** was formed alongside the major (*R*)-**2a** product (Table 1). For the LEHs, inversion of configuration at the benzylic carbon of styrene oxide hasn’t previously been shown, yet conforms to the proposed acid catalysed enzymatic mechanism, whereby attack of water on the epoxide primarily occurs on the more substituted carbon of the epoxide [42].

The *para-*fluoro and *para-*bromo styrene derivatives, **1b** and **1c,** were also accepted by all active LEHs and ⍺/β EHs with comparable yields to that of **1a** (Table 1). The LEH from pQR1980 and ⍺/β EH from pQR1984 had higher enantioselectivity for **1b** compared to **1a**, forming the (*R*)-diol in 22% *e.e.* and the (*S*)-diol in 33% *e.e.,* respectively (Table 1). The enzymes from pQR1986 and pQR1990 gave greater yields of the corresponding diol with **1c** when compared to **1a**, while the enzymes from pQR1980, pQR1982 and pQR1984 had lower conversions when the *para*-bromo substituent was present (Table 1). The enzyme from pQR1984 was particularly sensitive to the presence of the bulkier bromo-substituent as the yield of the diol dropped to 28% when this group was present compared to 61–86% when it was absent (Table 1). Overall, *para*-substituted substrates were tolerated by the active EHs.

Epoxides (**1d-g**) containing substituents on the C2 carbon were poorly accepted, suggesting low tolerance of groups at this position. The LEH from pQR1980 could tolerate methyl groups on C2 for both enantiomers of *trans*-methylphenyloxirane (**1d**), while the LEH from pQR1982 accepted the (1*R*,2*R*)-enantiomer and the ⍺/β EH from pQR1984 accepted the (1*S,*2*S*)-enantiomer (Table 1). Bulkier epoxides such as phenylglycidol (**1e**), *trans*-stilbene oxide (**1f**) and strawberry aldehyde (**1 g**) were not accepted as substrates by any of the EHs tested.

The cyclic epoxide, cyclohexene oxide (**3b**) was also a substrate for the enzymes from pQR1980, pQR1982, pQR1984 and pQR1990, with the enzymes from pQR1984 and pQR1990 de-symmetrising the epoxide to give the (1*R*,2*R)*-diol in 84–88% *e.e.,* respectively (Table 1, Figure S6). This is comparable *e.e.* to reactions using the ⍺/β EH from Sphingomonas HXN-200 [33], but lower than the EHs described by Zhao and colleagues [17]. The LEHs from pQR1980 and pQR1982 also accepted **3b**, with modest conversions, and slight de-symmetrisation, favouring the (1*R,*2*R*) and (1*S*,2*S*)-diol in 22% *e.e.* and 17% *e.e.*, respectively. The enzyme from pQR1982 preferentially formed the (1*S*,2*S*)-diol which differs to the preferred enantiomer formed with *Re*LEH, instead producing the same diol enantiomer as that for the recently reported, Tomsk-LEH and CH55-LEH [10]. The LEH from pQR1980 produced the same enantiomer as *Re*LEH but with greater enantiopreference at 22% (*ee*_p_) [10,36]. However, it had very low conversion for this substrate (Table 1). Interestingly from directed evolution experiments on *Re*LEH it was shown that highly (*R*,*R*)-selective enzymatic catalysts could be obtained from two mutations, I80V and L114F, while conversely highly (*S*,*S*)-selective catalysts were obtained through the mutations, I80Y and I116V [36]. Sequence analysis of pQR1980 and pQR1982 indicated that they both contain a valine at position 116, a glycine at position 80 and a phenylalanine and leucine at position 114 (numbering is for *Re*LEH) (Figure S2B). Therefore, as wild-type LEHs, the enzyme from pQR1980 could be considered to contain a (*R*,*R*)-selective residue at position 114, and the enzyme from pQR1982 a (*S*,*S*)-selective residue at position 116. These sequence differences may account for the greater selectivity of the enzymes from pQR1980 and pQR1982 compared to *Re*LEH for the particular diol product obtained; yet improved selectivities may be achieved by variations in sequence at alternative residues. The 5-membered cyclic *meso*-epoxide, **3a**, was not accepted by any of the EHs tested.

All three putative LEHs from pQR1980−1982 were active towards the limonene oxides (±-**7**), while the ⍺/β EHs from pQR1984, 1986 and 1990 were not. Both LEHs from pQR1980 and pQR1982 gave near quantitative conversions for (-)-limonene oxide (**7**), while the enzyme from pQR1982 maintained quantitative conversion towards (+)-**7** and the LEH from pQR1980 had a lower conversion (Table 1). This suggests that pQR1980 preferred (-)-limonene oxide to (+)-limonene oxide. For (+)-**7**, chiral GC analysis indicated that the enzyme from pQR1980 preferentially hydrolysed the *trans* isomer as indicated by the presence of the *cis* isomer after 20 h of reaction. This differs to *Re*LEH, which preferentially hydrolyses the *cis* isomer of (+)-**7**, instead being similar to Tomsk-LEH and CH55-LEH [10]. pQR1981 was also active towards these substrates but with very low percentage conversions.

For both *cis* and *trans* mixtures of (+)-**7** and (−)-**7**, the enzymes from pQR1980 and pQR1982 were enantio-convergent, forming di-axial (1*S*,2*S*,4*R*)-**8** and (1*R*,2*R*,4*S*)-**8**, respectively in high *e.e.* (Table 1). This enantioconvergence has also been observed for *Re*LEH and the thermophilic LEHs, Tomsk-LEH and CH55-LEH.

The enzyme from pQR1982 has a good substrate scope for a wild-type LEH, which is comparable to the recently characterised Tomsk-LEH and CH55-LEH, as it can accept styrene oxide (**1a**), its analogs (**1b-1d**), 1,2-epoxyhexane (**5**) and cyclohexene oxide (**3b**) with decent % conversions. The activity observed on the styrene oxide analogs (**1b-1d**) is interesting, as no other LEHs described to date have either been tested or shown to have notable activity on them. *Re*LEH has been tested on methylphenyloxirane (**1d**) yet activity was not accurately quantified and was < 0.25% of activity on its natural substrate, limonene oxide [8,43]. The enzyme from pQR1984 also displayed a wide substrate scope, modest percentage conversions and interesting enantioselectivity. As such, the LEH from pQR1982 and ⍺/β EH pQR1984 were characterised further.

### 3.4 Biochemical Characterisation of the enzymes from pQR1982 and pQR1984 with respect to *rac*-**1a**

**Fig. 5:**
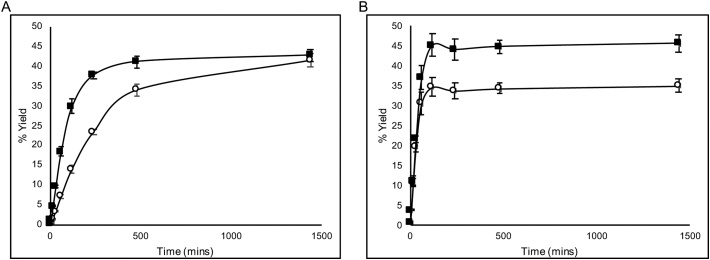
Reaction profiles of *rac*-styrene oxide (**1a**) hydrolysis. (A) – pQR1982, ○ – (*R*)-phenylethane-1,2-diol, ◼ – (*S*)-phenylethane-1,2-diol, (B) – pQR1984, symbols are the same as for (A). Yields were determined by chiral HPLC and are based on product formed. Assays were performed in triplicate and errors are < ± 3%.

**Fig. 6:**
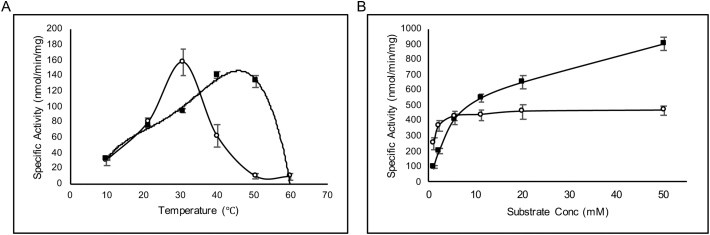
Temperature stability and substrate loading of the enzymes from pQR1982 (◼) and pQR1984 (○). (A) Temperature stability, assays were performed at least in duplicate. Trendlines are included only as a visual aid. (B) Substrate loading. Assays were repeated in triplicate.

**Fig. 7:**
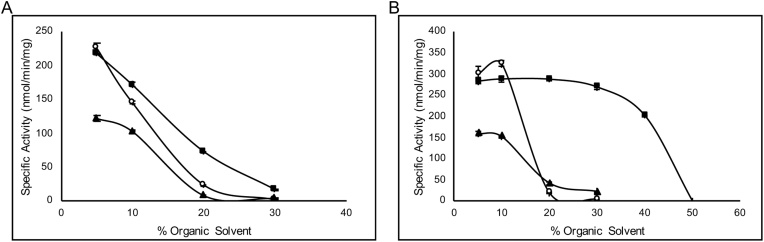
Organic solvent tolerance. (A) pQR1982, ◼ – MeOH, ○ −CH_3_CN, ▲ – TBME, (B) pQR1984, symbols are the same as for (A). Assays were repeated in duplicate.

To characterise the enzymes from pQR1982 and pQR1984, *rac*-**1a** was used as both of these enzymes displayed high conversions on this substrate. The reaction profile, temperature stability, substrate loading and organic solvent tolerance of the two enzymes were determined (Fig. 5, Fig. 6, Fig. 7). A time-course assay of the two enzymes indicated that both displayed preferential formation of the (*S*)-diol. However, the kinetics of formation differed, in that the LEH from pQR1982 formed the (*S*)-diol at a faster rate than the (*R*)-diol (Fig. 5A), while the ⍺/β EH from pQR1984 formed both diol enantiomers at an equivalent rate, but the (*S*)-diol plateaued at a higher yield (Fig. 5B). Overall, the enzyme from pQR1984 reached maximal conversion for *rac*-**1a** after 2 h, while the enzyme from pQR1982 reached it after 8 h of reaction. Combined with the regioselectivities of the two enzymes (Table 1), the LEH from pQR1982 appears to preferentially hydrolyse (*R*)-**1a** compared to (*S*)-**1a**, as it inverts the configuration at the benzylic carbon and the (*S*)-diol is formed at a faster rate compared to the (*R*)-diol. However, the ⍺/β EH from pQR1984 displayed little preference for hydrolysis, and the imperfect terminal regioselectivity for formation of the diol from (*R*)-**1a** (Table 1), could account for the enrichment of the (*S*)-diol product.

Temperature stability was assessed from 10 to 60 °C and indicated that the ⍺/β EH from pQR1984 had a narrow temperature optimum of around 30 °C, while the LEH from pQR1982 was active at a broader temperature range with activity peaking between 40–50 ºC (Fig. 6A). The LEH from pQR1982 had thermostability comparable to that of *Re*LEH and Tomsk-LEH but lower than that of CH55-LEH. However, the LEH from pQR1982 was isolated from a mesophilic *Rhodococcus* strain while CH55-LEH came from a hotspring, which accounts for the unusually high temperature tolerance of CH55-LEH. Nevertheless, the LEH from pQR1982 had comparable temperature tolerance to the reported LEHs. The ⍺/β EH from pQR1984 had a temperature optimum of 30 °C which is within the typical reported temperature range of 15−50 °C for ⍺/β EHs [32,41]. To determine if lower enzyme and higher substrate concentrations could be used, the enzyme concentration was reduced to 0.1 mg/mL from 0.4 mg/mL and *rac*-**1a** was added at concentrations of 1 mM – 50 mM. After 30 min of reaction it was evident that the ⍺/β EH from pQR1984 reached saturation at a substrate concentration of 5−10 mM, while the LEH from pQR1982 appears to tolerate higher substrate concentrations, not reaching saturation even at 50 mM of *rac*-**1a** (Fig. 6B).

Biocatalytic reactions using EHs have been conducted in the presence of organic solvents, as the epoxide substrates tend to have low solubility in water. In some cases, the organic solvent present has reached high levels of 25–40% [4,22,23]. Therefore, it was of interest to determine the organic solvent tolerance of the two most active enzymes. This was evaluated against three solvents; acetonitrile (CH_3_CN), methanol (MeOH) and *tert*-butyl methyl ether (TBME). These were selected as CH_3_CN is commonly used in epoxide hydrolase assays as a co-solvent, and MeOH and TBME represent more sustainable green solvents. At 10 mM of *rac*-**1a**, the enzymes from pQR1982 and pQR1984 tolerated the presence of MeOH better than CH_3_CN and TBME (Fig. 7). Complete loss of activity was observed for both enzymes with around 30% of TBME and CH_3_CN. However, the ⍺/β EH from pQR1984 had greater tolerance towards MeOH, having comparable activity between 5–30% MeOH and complete loss of activity at 50% MeOH. The LEH from pQR1982 was less tolerant than the ⍺/β EH from pQR1984 for all solvents tested.

## 4 Conclusion

In conclusion, through sequence analysis of several soil bacterial genomes, twenty-nine putative EHs were identified. From these, eight were functionally characterised, with five being shown to be active EHs. The enzymes from pQR1982 and pQR1984 showed wide substrate acceptance, and with respect to styrene oxide (**1a**), catalysed hydrolysis with alternative regioselectivities. This is of value as from the same enantiopure epoxide, access to both (*R*)- and (*S*)-diols can be obtained. The LEHs from pQR1980 and pQR1982 showed activity towards para-substituted styrene oxide derivatives. These haven’t previously been tested as substrates for LEHs nor shown to be accepted by such enzymes.

The de-symmetrisation of *meso*-epoxides such as cyclohexene oxide, is of particular interest as these reactions can proceed with a theoretical yield of 100% and reports of such bio-transformations with wild-type enzymes are scarce [4,17,33]. In this study we have identified two enzymes from pQR1984 and pQR1990 which can de-symmetrise cyclohexene oxide (**3b**), furnishing the (*R,R)*-diol with high enantiomeric excesses of 84% and 88%. The LEHs from pQR1980 and pQR1982 were also capable of de-symmetrising this substrate with greater *ee_p_* than *Re*LEH, and the LEH from pQR1982 had the same enantiopreference as Tomsk-LEH and CH55-LEH.

Biochemical characterisation of the enzymes from pQR1982 and pQR1984 indicated temperature optima comparable to those reported for their respective enzyme families. The enzyme from pQR1984 could tolerate high levels (30–40%) of the green solvent, MeOH. This is of interest as the usage of a low-cost co-solvent to solubilise the epoxide substrate is often necessary and more economical when scaling-up the reaction. The enzymes found can be useful starting points for future directed evolution experiments to generate novel EHs for bio-industrial applications.

## CRediT authorship contribution statement

**Gorjan Stojanovski:** Methodology, Validation, Investigation, Project administration, Writing - original draft, Writing - review & editing. **Dragana Dobrijevic:** Methodology, Supervision, Investigation, Writing - review & editing. **Helen C. Hailes:** Conceptualization, Supervision, Writing - review & editing. **John M. Ward:** Conceptualization, Supervision, Resources, Funding acquisition, Writing - review & editing.

## Declaration of Competing Interest

None.
