# Supplementary material for: Identification and catalytic properties of new epoxide hydrolases from the genomic data of soil bacteria
Source: Enzyme Microb Technol. 2020 Sep;139:109592. doi: 10.1016/j.enzmictec.2020.109592 (PMC7429986; doi:10.1016/j.enzmictec.2020.109592)
Supplement: Supplementary file 5 [file mmc5.docx]

Supplementary Material

General Methods

^1^H and ^13^C nuclear magnetic resonance (NMR) spectra were recorded at 700 MHz and 175 MHz respectively on a Bruker Avance Neo 700 instrument in either deuterated chloroform (residual solvent peak, ^1^H: 7.26 ppm, ^13^C: 77.3 ppm) or deuterium oxide (residual solvent peak, ^1^H: 4.79 ppm, ^13^C: None). For ^1^H spectra, chemical shifts are quoted to the nearest 0.01 ppm, with signal splittings reported as: singlet (s), doublet (d), triplet (t), quartet (q), multiplet (m) or combinations thereof. For ^13^C spectra, chemical shifts are reported to the nearest 0.1 ppm. Mass spectrometric analysis was performed on an Agilent LC system containing a C-18 column connected to an Agilent 6510 Q-TOF mass spectrometer with electrospray ionisation (ESI) and the Waters LCT Premier XE ESI Q-TOF mass spectrometer in the Department of Chemistry, UCL.

Data Analysis

Enantiomeric excess values were calculated from HPLC/GC traces using the following formula:

$${ee}_{p}= \frac{(A-B)}{(A+B)} \times100$$

Where *A* and *B* are either the (*R*) or (*S*) enantiomer of the product diol, dependant on which is in excess. The same formula was used to calculate the diastereomeric ratios for reactions of substrates (1*R*,2*R*)-**1d**, (1*S*,2*S*)-**1d** and the limonene oxides (±-**7**).

General Preparation of 1,2-diol standards

Non-commercially available diol products were accessed via the method described in [1]. In brief, approximately 2 mmol of epoxide was added to a stirred solution of H_2_O (15 ml) or a 1:1 mixture of H_2_O:1,4-dioxane (30 ml) and heated at 60 ºC under reflux overnight. Reaction progress was monitored by TLC. The products were extracted with 3 x EtOAc (15 ml) and the combined organic layers were dried over Na_2_SO_4_, filtered and the solvent removed *in vacuo*. Diol products were either pure at this point or were purified further by silica column chromatography.

1-(4-fluorophenyl)ethane-1,2-diol (**2b**)

Title compound was obtained as a white powder and 1:1 mixture of enantiomers (86% yield). ^1^H NMR (CDCl_3_; 700 MHz) δ 7.34-7.30 (2H, m, Ar-H), 7.06-7.02 (2H, m, Ar-H), 4.79 (1H, dd, *J* = 8.4, 3.4 Hz, 1’-H), 3.72 (1H, dd, *J* = 11.4, 3.4 Hz, 2’Ha), 3.61 (1H, dd, *J* = 11.4, 8.4 Hz, 2’-Hb), 2.71 (2H, brs, OH); ^13^C NMR (CDCl_3_; 175 MHz) δ 162.6 (d, ^1^*J*_CF_ = 246.0 Hz, C-4), 136.3 (d, ^4^*J*_CF_ = 3.0 Hz, C-1), 127.9 (d, ^3^*J*_CF_ = 8.1 Hz, C-2, 6), 115.6 (d, ^2^*J*_CF_ = 21.4 Hz, C-3, 5), 74.2 (C-1’), 68.2 (C-2’). Spectral Data are in agreement with literature reports [2].

1-(4-bromophenyl)ethane-1,2-diol (**2c**)

Title compound was obtained as a white powder and 1:1 mixture of enantiomers after silica column chromatography (0-60% EtOAc in petroleum ether 40-60 ºC) (28% yield). ^1^H NMR (CDCl_3_; 700 MHz) δ 7.49 (2H, d, *J* = 8.4 Hz, Ar-H), 7.24 (2H, d, *J* = 8.4 Hz, Ar-H), 4.78 (1H, d, *J* = 7.8 Hz, 1’-H), 3.77-3.70 (1H, m, 2’-Ha), 3.63-3.58 (1H, m, 2’-Hb), 2.70 (1H, brs, OH), 2.16 (1H, brs, OH); ^13^C NMR (CDCl_3_; 175 MHz) δ 139.6, 131.8, 127.9, 122.0, 74.2, 68.0. Spectral Data are in agreement with literature reports [2].

(1*RS*,2*RS*)-1-phenylpropane-1,2-diol (**2d**) - (from (1*S*,2*S*)-**1d**)

Title compound was obtained as a white powder and 1:1 mixture of enantiomers after silica column chromatography (0-60 % EtOAc in petroleum ether 40-60 ºC) (55% yield). ^1^H NMR (CDCl_3_; 700 MHz) δ 7.37-7.28 (2 x 5H, m, Ar-H), 4.67 (1H, d, *J* = 4.4 Hz, 1’-H), 4.36 (1H, d, *J* = 7.4 Hz, 1’-H), 4.02-3.98 (1H, m, 2’-H), 3.88-3.83 (1H, m, 2’-H), 2.42 (4H, brs, OH), 1.08 (3H, d, *J* = 6.4 Hz, 3’-H), 1.05 (3H, d, *J* = 6.3 Hz, 3’-H); ^13^C NMR (CDCl_3_; 175 MHz) δ 141.2, 140.5, 128.7, 128.5, 128.3, 128.0, 127.0, 126.8, 79.6, 77.7, 72.4, 71.4, 18.9, 17.4; ES^+^-HRMS: *m/z* Calculated for C_9_H_13_O_2_: 153.0916, Found: 153.0915 [M + H]+. Spectral Data are in agreement with literature reports [1].

(1*RS*,2*RS*)-1-phenylpropane-1,2-diol (**2d**) - (from (1*R*,2*R*)-**1d**)

Title compound was obtained as a white powder and 1:1 mixture of enantiomers after silica column chromatography (0-60% EtOAc in petroleum ether 40-60 ºC) (59% yield). ^1^H NMR (CDCl_3_; 700 MHz) δ 7.38-7.28 (2 x 5H, m, Ar-H), 4.67 (1H, d, *J* = 4.4 Hz, 1’-H), 4.37 (1H, d, *J* = 7.4 Hz, 1’-H), 4.02-3.99 (1H, m, 2’-H), 3.88-3.83 (1H, m, 2’-H), 2.40 (4H, brs, OH), 1.07 (3H, d, *J* = 6.4 Hz, 3’-H), 1.05 (3H, d, *J* = 6.3 Hz, 3’-H); ^13^C NMR (CDCl_3_; 175 MHz) δ 141.2, 140.5, 128.7, 128.5, 128.3, 128.0, 127.0, 126.8, 79.6, 77.7, 72.4, 71.4, 18.9, 17.4; ES^+^-HRMS: *m/z* Calculated for C_9_H_13_O_2_: 153.0916, Found: 153.0916 [M + H]+. Spectral Data are in agreement with literature reports [1].

1-phenylpropane-1,2,3-triol (**2e**)

Title compound was obtained as a colourless oil and a mixture of diastereoisomers after silica column chromatography (0-15 % MeOH in DCM) (29% yield, 58:42 mixture). ^1^H NMR (D_2_O; 700 MHz) δ 7.42-7.33 (10H, m, Ar-H), 4.64 (1H, d, *J* = 6.4 Hz, 1’-H), 4.61 (1H, d, *J* = 7.1 Hz, 1’-H), 3.90-3.86 (1H, td, *J* = 7.0, 3.3 Hz, 2’-H), 3.86-3.82 (1H, td, *J* = 6.8, 3.7 Hz, 2’-H), 3.77 – 3.74 (1H, dd, *J* = 11.8, 3.3 Hz), 3.60-3.56 (1H, dd, *J* = 11.8, 6.8 Hz), 3.50-3.47 (1H, dd, *J* = 11.8, 3.3 Hz), 3.39-3.35 (1H, dd, *J* = 11.8, 6.9 Hz); ^13^C NMR (D_2_O; 175 MHz) δ 141.1, 140.9, 129.2, 129.1, 128.8, 128.7, 127.7, 127.3, 76.0, 75.2, 74.8, 74.5, 63.2, 63.1; ESI^+^-MS: *m/z* 191 [M + Na]+. Spectral Data are in agreement with literature reports [1].

(+)-limonene-1,2-diol [(+)-**8**]

Title compound was obtained as a colourless oil and a mixture of (1*S*,2*S*,4*R*) and (1*R*,2*R*,4*R*) diastereoisomers in an 8:1 ratio (76% yield). ^1^H NMR (CDCl_3_; 700 MHz) δ 4.73 (2H, m, 1’-H_2_), 3.64 (1H, m, 2-H), 2.26 (1H, m), 1.95-1.90 (1H, m), 1.73 (3H, s, 3’-H_3_), 1.69-1.65 (1H, m), 1.58-1.51 (6H, m), 1.26 (3H, s, 7-H_3_); ^13^C NMR (CDCl_3_; 175 MHz) δ 149.4, 148.6, 109.2, 109.1, 74.1, 74.0, 71.4, 43.8, 38.7, 37.6, 36.2, 34.2, 33.8, 28.8, 26.8, 26.3, 21.2, 21.1, 19.0; ESI^+^-MS: *m/z* 193 [M + Na]+, 171 [M + H]+, 153, 135. Spectral Data are in agreement with literature reports [1].

(-)-limonene-1,2-diol [(-)-**8**]

Title compound was obtained as a colourless oil and a mixture of (1*R*,2*R*,4*S*) and (1*S*,2*S*,4*S*) diastereoisomers in an 9:2 ratio, (51% yield). ^1^H NMR (CDCl_3_; 700 MHz) δ 4.73 (2H, m, 1’-H_2_), 3.64 (1H, m), 2.26 (1H, m), 2.16 (2H, s), 1.72 (3H, s, 3’-H_3_), 1.26 (3H, s, 3’-H_3_); ^13^C NMR (CDCl_3_; 175 MHz) δ 149.5, 148.6, 109.2, 109.1, 74.1, 74.0, 71.4, 43.7, 38.7, 37.6, 36.2, 34.2, 33.8, 28.8, 26.8, 26.3, 21.2, 21.1, 19.0; ESI^+^-MS: *m/z* 193 [M + Na]+, 171 [M + H]+, 153. Spectral Data are in agreement with literature reports [1].

Supplementary Figures

**Figure S1**: Heatmap of multiple sequence alignment of identified EHs, colouring is based on total percent identity from 0 – 40%. Average between-group sequence identity: ⍺/β EHs (- EH-N): ⍺/β EHs (+ EH-N) = 18%, ⍺/β EHs (- EH-N): LEH = 9%, ⍺/β EHs (+ EH-N): LEH = 14%, Average within-group sequence identity: ⍺/β EHs (- EH-N) = 29%, ⍺/β EHs (+ EH-N) = 40%, LEHs = 26%.

Figure S2: Multiple sequence alignments of (A) ⍺/β EHs and (B) LEHs. All functionally relevant residues are marked with asterisks, the catalytic residues are highlighted in red, with green/orange boxes indicating other functionally relevant motifs/residues specific to either ⍺/β EHs or LEHs.


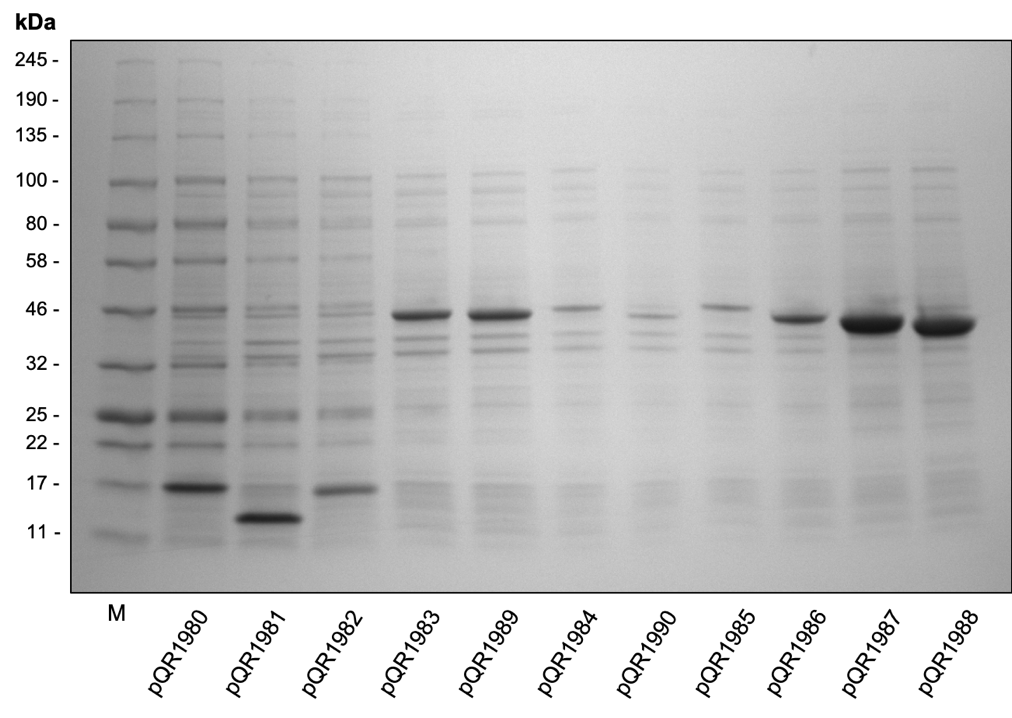


(Figure S3: SDS PAGE analysis of protein expression, Whole cell fractions are shown.)


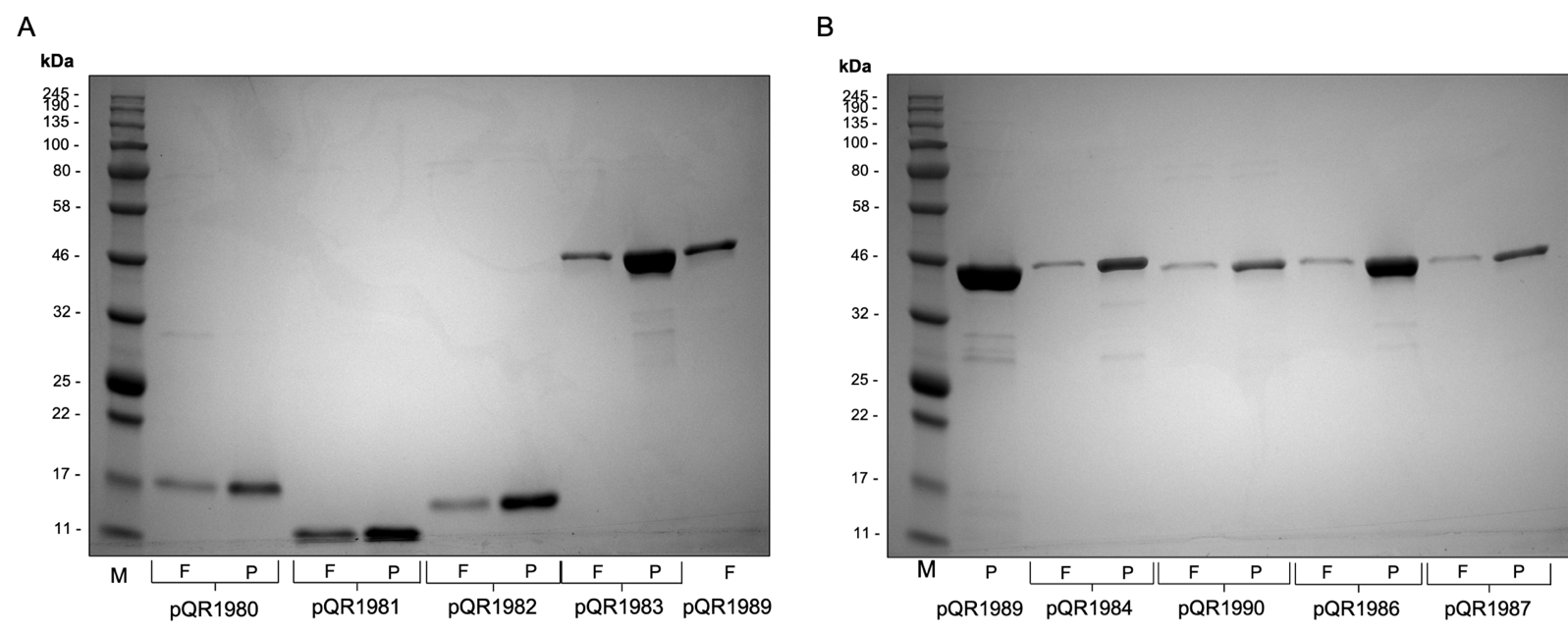


(Figure S4: SDS PAGE analysis of purified proteins, F - the flow-through fraction from Ni^2+^-NTA elution, prior to elution of each protein. P – the protein fraction from Ni^2+^-NTA elution.)


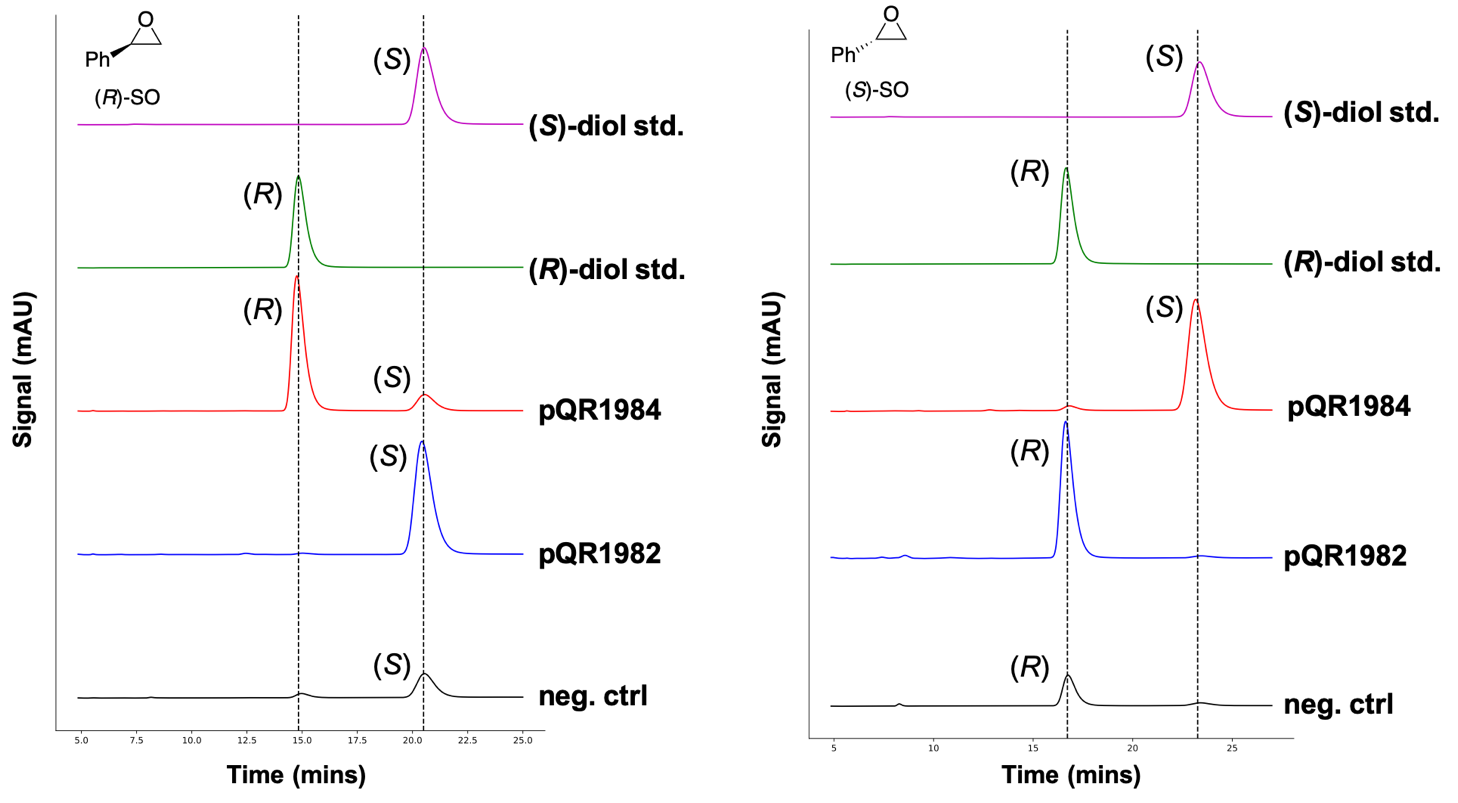


(Figure S5: Chiral HPLC traces of (*R*)-**1a** and (*S*)-**1a** for pQR1982 and pQR1984.)


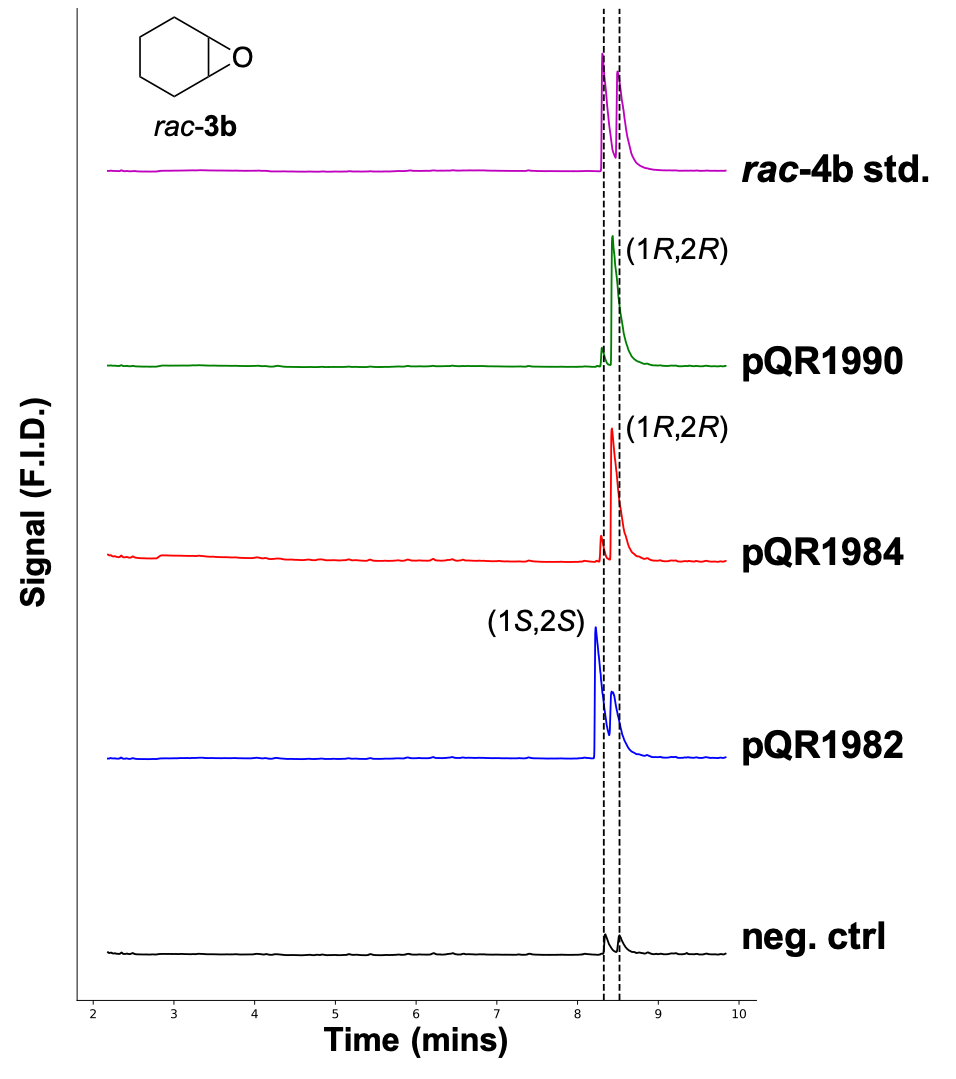


(Figure S6: Chiral GC traces of *rac*-**4b** for pQR1982, pQR1984 and pQR1990.)

Protein sequences of characterised and putative EHs

**pQR1980**

MNTNSHAPTGTDRTETADIQVVRAFLDALVAGDPDTAQQYLHDDIIWHNVSLPKIRGIGAVMRIFRGMSRPGFGFDVRLHHIAGDGTAVLTERTDVLIYKRLRSEFWVCGTFEMRDGKIAVWRDYFSNRDVLWGTLKGIVRAF

**pQR1981**

MSNSDVLLKLFDRWNTDFDTMCEATREAFAPDCIWENAGMPAVVGFAEAKEKILLPSHAGPLYMESIRVDTLHIAEIDNVVYSERIDFINRARGLPVIDIKIAGFVEFDGEGRIAHWRDYCDPAVLHHL

**pQR1982**

MTPDQLVTQFCAEWIEPDPAKIAEYFAEDAVYHNIPMEPVIGRDAIREFVAGFTAAFGGIDFRVHRQVTDGGQSPNAEESSGVVMNERIDVFTLNGTVVELPVVGVFEITGGKITTWRDYFDMAPIQAATGGG

**pQR1983**

MSVTSEVAIRPFTIEFPETELEALRQRIAATRWPDKETVADHSQGVPLATSQALAQYWASNYDWRKVEAKLNALPQFITEIDGLDIHFIHIRSKHEDALPLIVTHGWPGSIIEQLKIIEPLTDPTAHGGDASDAFHLVIPSGPGYGFSGKPTTTGWGLDRIARAWIELMKRLGYTRYAAQGGDWGAIVTELMALQAPPELVGIHTNMASVVPPEIDQALRTGNPLPSDLSDDEKAASDQLAFFYQHIGYAVEMGTRPQTLTGLTDSPVGLATFMLDHDAKSLELIARAFAGQPEGLTRDDVLDNITLFWLTNTAVSAARLYWELTSPAFLATRGVSIPVAVSVFPDELYQAPRSWTKKAYPNLVHYNKLDKGGHFAAWEQPQLLAEEIRVGLRSLR

**pQR1984**

MPRPTSDVHAFEAHAPDADLDDLRARLAAARLPEAETVYRAAPGPRRWEQGVPLADLVDVVDYWRTGYNWRSFEERLNRIGQFRTTVDDLGIHFLHRRSARADATPLVLTHGWPGSIAEFVDVVDELADPKDADAPAFHVVVPSLPGFGYSDKPATTGWGTEKIAAAWVELMGRLGYSKFAAHGGDWGGNITTVLGGRFPAHVLGIHTLFAEAPPGLTTDGLTAVERKWTEETRDFWHHRAAYAKQQATRPQTIGYSLVDSPVGLLAWILDKFAEWSDTEDSPFETMSRDRILDDVTLYWLTRTGASAARIYYESHNSLDPELRVDVPSAITMYPRDIEKCPRPWAQERYRHIVRWRSPETGGHFPSLEVPEYFVKDLQEGLAAVLAANR

**pQR1985**

MNSHFSEEVSESYWLALRRVMEPEKFNIHIPDERINDLRQRLRATNWPGDFGNSDWDYGVEQGWLRDMVSYWADDFDWRAAEARMNAYDQFRVEIDGVLIHFMHIRSGRPNAIPLILTHGWPWTFWDWRGVVEELTRDNDDVAFDIVVPSLPGYGFSSPLRIPGLDIRGIGRLWVKLMTEVLGYERFAAAGGDLGSAITAELGHAYPERMIAVHFCILLLPEFHYKDVRQEDYAPDEKWMIARNREAVPLITAHRVLHSTEPQTISYALADSPVGTAAWIWARRRAWSDSNSDVMSYRGRDFLCSLASVYWLTNTIGSSLRLYKEQYGGGVRATGMPAPTEWSLLRQDAPSIPVPTGVAVAPKEVALMPRAAVERRVNLARWTVLPRGGHFLPSEAPDLLAQEYRAFFGEHRLSI

**pQR1986**

MTDTTKSIRPFRIDIAQAQLDDLAARLARTRFGTPLPGDDWKTGVPTSYLVELVAYWRDEFDWRAQEKSLNTFPQFVTEIDGQNIHFLHVRSPEPDALPLVLTHGWPGSFVEFLDVIGPLTDPVAHGGNPADAFHVVVPSLPGFGFSGPVRESGWNIHRIAGAWAELMSRLGYDRYGVQGGDIGAGVSPEVARIARDAVAGVHLNGNIGVPVHDVDEAERASLTPLERDRLDRVLRFLTDEFGYISIQSTRPATLGAALADSPVGQLAWIVDKFREWTHPREALPHDVVGIDRLLTNVMLYWLTDTASSAAYVGYMQESSWGTANSVSGIPTAVIVFAHDVGIRRYAEREHAITRWTDVEDRGGHFAALEEPATLTADIRDFFAGLR

**pQR1987**

MSTSKPFTLRIPDAAIADLRERLARTRFPDEPPLAPWSTGTSVAYLKSLTDHWQNDFDWRAWEAKLNSFPQFTLPVRGIEVHFIHAPSKKPGAMPLLLSHGWPGSVFEFHKIIPLLTEHFTVVAPSLPGYTLSFTPGQQRLGLVEIAEVFAELMTALGYERFGAQGGDWGSFITSVLGHRFAARTSGIHLNMLPLRRDLKMPPNPNTEERMYLDQLDNFVKEETGYQWIQGTRPQTLAFGLTDSPAGLAAWIVEKFHAWTDNNGNPEDAVTRDEMLANISLYWFTGAIGSSFWPYYARMHSPWPVPEGEIVAAPMGYAEFPRENLTPPRSLAEQSFGDIRRWTRMTKGGHFAALEQPQALAEDIIAFFGALE

**pQR1988**

MPDITPFKIAIPESELTELKARLANTRLANEIPGAGWSYGMASGFVTRAIERLRNGFDWRAEEATINSHPQFVTEIDGQTIHFLHVKSPAANAVPLLLLHGWPSSFVEFLGAIGPLTAAGFDLVIPSLPGFGFSGPTREAGWNDGRIANAMLELMSRLGYRRFGVQGGDAGAIIAPAIGRAAPDRVIGVHVNAATMGFIPLGLTDPSEVATFSDAEKSRLQRLQQFMAERFGFNALQSSRPQTLAYGITDSPAGLIAWLGDMFAGFGDSPDAVEQDTFLTNVLVYWFTGTAASSIRLYYENAHDPDAWSPKPNSGVPTGVAVFARDEVAIRRYGEEGNTIVCWTELASGGHFAAMEVPEVWAGEVAAFFTRLRG

**pQR1989**

MDNPQAFSIDIPQTRIDDLYRRLDDTRWPDMLPGAGSTSGAPLHRIQELATYWRNTFDWRRAEKRLNTYPQFRTEIDGTTIHYVTVRSPREDAKPLLLIHGWPGSYLEYLDTVGPLIDPEAHGGSASDPAFHVVLPSLPGYTFSGPVAEPGWGSERVARALTTLMTRLGHDTFMVHGGDWGERIGRDVAKQAPDRVVGVHITTFFCSPPFAGGVGELTPEEQALLDDYLTRDFSVGYFHLLSERPQTVGYGWSDSPVSLLAWMTEKFDAWTDWTNSPEEAVDVDTFLTNVSMYWFTNTANSAGRIYFEEARSGTDRTSDFNATPTGCGIYPREMSVPIRSLVEKTNNVVFWAEHDRGGHFAALEEPDLVIEDLRAFSRILAAR

**pQR1990**

MAVRPYTIAVPEEVLVDLKERLARTRWPEKIEGAGWDYGTDVAYLRELCDHWAHRYDWRDWERRLNSIPGYVCEVDGVDLHFWHVRGTGPNPTPLLLLHGWPGSIVEFLELIGPLTDPAAHGGDPADSFDVVVPELPGFGFSGAPRTAGWCLSHIASVFNSLMVDQLGYDRYAVQGGDFGGLIASRMGAYHSESVLGIHLNLIMGTPPAEPGPEDLEHLERYSRFSERALGYLQVQNTTPDSLMIAQSDSPAGLASWIIEKFRSWSDCGGDIESSFSKDLLLTNLMFYWAPNSAASAARLYYETSRAEDWYLKARVELPVAYAEFPHENFVPPRTWVDTQYDIARWTTMPSGGHFAALEEPHLLLDDVRTYFRSLR

**EH12**

MILDRLCRGLLAGIALTFSLGGFAAEEFPVPNGFESAYREVDGVKLHYVKGGQGPLVMLVHGFGQTWYEWHQLMPELAKRFTVIAPDLPGLGQSEPPKTGYSGEQVAVYLHKLARQFSPDRPFDLVAHDIGIWNTYPMVVKNQADIARLVYMEAPIPDARIYRFPAFTAQGESLVWHFSFFAADDHLAETLIAGKERFFLEHFIKSHASNTEVFSERLLDLYARSYAKPHSLNASFEYYRALNESVRQNAELAKTRLQMPTMTLAGGGHGGMGTFQLEQMKAYAEDVEGHVLPGCGHWLPEECAAPMNRLVIDFLSRGR

**EH13**

MLTPVASSHSSDPRPPGVELRRVAVNGVELDVALQGEGPAVLLLHGFPHTWQLWTRVMGPLAEHHRVVAPDMRGAGASTRAADGYDAGTLACDAEALLEALGVRSADVVGIDAGTPPAFLLAMCRPDLVRRLVVMESLLGGLPGAEEFLARGAPWWFGFHAVPGLAETVLTGHEARYVDWFLDAGSLGEGVDPAVRDAFVRAYTGTEALRCAFSYYRALPTSGEQIAQAAAGGRLTVPTMAVGARPVGRALERQLRPVADHLVGHVIEDCGHIIPLHRPEGLLRLLEPFLAAGGTPSGAPMISGPAGSR

**EH14**

MMFDHDGTPVRTGRAAVNGTSLHYRAAGSGPAVVLLHGVPKTSYHWRHLVPKLTPHYTVVAPDLRGLGDSARPADGYDSATMSDDIAELMNHLGHESYAVVGEDWGAVIGYQLAARHRDRVTALVFAEALFPGFGFEDHTALTAENVAGGMHLWHLGFYFQPDIPEMLIAGHERELITYMIKFERSRPDSATPEAIDEYVRCYSMPGGIRAMLAVYRAMLVDAEQNRRAARKKLDIPVLALGGSAFIGDRNESQMRLMAHDVTGHVFDAGHDLAEEVPDEMADVLLPFLATHQ

**EH15**

MPQPPVTDQQTGERDTRQDGRPGQAAGRPVHRLVPSPAGRIHLVEQGSGPLVLLVHGFPESWYSWRHQLPALAAAGFRAVALDVRGYGRSSRPDAVEAYRMLDLVADNVAVVEALGESSAVVVGHDWGANIAAHSALLRPDVFRAVGLLSVPYTPPGGPRPSEAFAGMSDPAGPFAGQEFYVSYFQEPGRAEAEIEPDVRGWLAGLYAALSAGTMPGPQDPDPHFVAPGGRMRDRFPSAGRLPSWLTEEDLDVYAGEFERTGLTGALNRYRNMDRDWADLAAHEGAPITQPSLFLGGALDASTTWLSDAIEAYPVTLPGLSASHLLDGCGHWLQQERPEETNRLLTEWLTGLPS

**EH16**

MTGSSTSPGQSPSPQHPNPTSVVRLGIPGGPEVTHRDVAANGARFHIAELGDGPLVLLLHGFPQFWWTWRHQLVALADAGFRAVAMDLRGVGGSDRTPRGYDPAGLALDITGVIRSLGEPDAALVGHDLGGYLAWTAAAMRPKLVRRLAVSSMPHPRRWRSAMLGDVRQSRAGSYVWGFQRPWVPERQLTADDGALVGRLLHDWSGPRLLDDDAVTAYRRAMCIPSTAHCSVEPYRWLVRSLARPDGIQFYRRMKRPVRVPTLHLHGSLDPVMRTRSAAGSGQYVEAPYRWRLFDGLGHFPHEEDPVAFSTELINWLKDPEPDR

**EH17**

MTDDATAPSSSCDQNPVPGLPLHDLAGFTHRWVDAEGIRLHAVEGGRPAGPTVVLLAGFPQTWWAWRKVMPGLAARFRVIAIDLPGQGHSERPRGGYDTHTVASRVQTALTALDVPKYWLVGHDVGAWVAFSLALKYEERLHGVALLDAGIPGITLPDSIPTDPDRAWKTWHFAFHLVPELPETLLTGRERDYVDWFLKVKTLSPDTFDGAEIDHYAAAVAAEGGLSASLAYYRDAAESARRNHDALERGHLTVPVLGVSGSHGSIPDMAASIGPWAANATGAVIPQAGHFIPDEQPEATVKVLTAFIDYERAE

**EH18**

MPVTAAHHLRRIVTNGVQLNVAIAGDGPAVLLLHGFPHTWQLWSGIMGRLARQYRVIAPDLRGFGASARAVEGYDAGTLAADAEGLLDALGEPSAAVVGIDAGTAPAVLLALRRPGLVRRLVVMEALLGRLPGAEHVVAGGAPWWFGFHAVPGLAETVLAGNETQYIDWFLDSGTLGRGVPDDVRAAFVHAYTGSEALRCAFSYYRALPTSAQQIQDAVATARLTMPTMAVGSHPVGTGLERQLRPIADDLVGHHLQDCGHIIPLDRPDALFALLAPFLSADLPK

**EH19**

MPRPTSDVQAFEAHATDADLDDLRARLAAARLPEAETVHRAPPGRRRWEQGVPLADLVDVVNYWRTGYNWRSFEERLNQIGQFRTTIDDLGIHFLHRRSARADATPLLLTHGWPGSIAEFVDVVDELADPKAADAPAFHVVAPSLPGFGYSDKPTATGWGTERIAAAWVELMERLGYREFVAHGGDWGGNITTVLGGRFPAHVLGIHTLFAEAPPGLTADGLTAVERRWTEETHDFWRHRAAYAKQQATRPQTIGYSLVDSPVGLLAWILDKFAEWSDTDDSPFETISRDRILDDVTLYWLTRTGASSARIYYESHNSLDPELRVDVPSAITMYPRDIEKSPRPWAQERYRQIVRWTEPEIGGHFPSLEVPEYFVRDLQEGLAAVLAAKR

**EH20**

MLPEPRFVATNGVRLAVFEAKPERRTKDVCVVLCHGFPELAASWRKQLQPLADAGFHVMAPDMRGYGRSSGPEDRRAYSIAETTADVAGLIADAGYEKAVVVGHDFGGMVSWMMPHLQPDSVAGVITLNTPFGHSRENPVDRYAQLYGPRNYVAHFQTRECEQKLDEDPERSFRFFMRRDTGSGTNLSRTGRHDPDSMGYIHWLWDDESTWPGEVLMTPAELRYYADAYTRTGFRGGLSWYHSILRNWEVQTALFPDGNVPKVDVPALLIAARHDPICHPLLTDDLLRYFAEFERRMIDTGHWTQLEDPEGTNALLLDWLNRHF

**EH21**

MTAPDSTASVVRIGLPDGTEVTHRDVAANGARFHIAEVGDGPLVLLLHGFPQFWWTWRHQLPALADAGFRAVAMDLRGVGGSDRTPRGYDPANLALDITGVIRSLGEPDAALVGHDLGGYLAWTAAVMRPKLVRRLAVSSMPHPRRWRAAMLADVRRSSHIWSFQRPWLPERALTADDGALVGRMIRDWSGPQLPADETVEVYQRAMAIPSTAHCSIEPYRWMVRSMARPDGLQFNRRMKLPVRVPTLHLHGSADPVMRTRSAAGSGEYVEAPYRWRLFDGLGHWPHEEDPTAFSTELINWLKDPEPDR

**EH22**

MHQPAPSADLSHRTVEAPAGRLHLVEQGTGPLVLLVHGFPESWYSWRHQLPALAAAGYRAVALDVRGYGRSSKPAAPDAYRMLDLVADNVALVRALGEERAVVVGHDWGSNIAATSALLHPEVFRAVGLLSVPYAPPGGPRPTDAFAQMGGDEEFYVSYFQQPGRAEAEMEPDVRGWLAGFYAALSADTMPAPGEPDPHFVARAGGRLRDRFPGGKPPAWLTEDDLDVYAGEFERTGTTGALNRYRNMDRDWEDLAPYRGAPITQPSLFIGGALDASTTWMADAIDAYPATLPGLAASHILAGCGHWIQQERPEEVNRLLIAWLATLNG

**EH23**

MTTAIKHHDVEVNGVRLHVAEQGEGPLVVLLHGFPECWYSWRHQFAPLAAAGYRVVAPDQRGYGRSDRPKDPSQYTVHHLTGDVVALIHALGESRAFLVGHDWGAPVAWTTAQLRPDLVRGVAGLSVPPFPRGRQSMAAAYRQQLGERYYFVYFQEPGRADAELARDVRTTFRRILAGRPADGDGLLWNAPADGRLLDALADPGELPAWLTEADLDAFVAEYAPHGAEAFTGGLNWYRATDLNWELTAALDGLPVTVPALFLAGEQDIVTALPAARDFIAALPQVLPRLHHSALLPDCGHWTQQERPDEVNAALLDFFGSVRD

**EH24**

MIDVNGIRLHIAEEGEGPLVVLLHGFPESWHSWRHQFGPLAAAGFRVVAPDQRGYGRSDHPEDVAAYSILHLVGDVIGLIHALGEREAFVVGHDWGAPVAWHTALLRPDVVRGVAGLSVPPPFRGERPPLAAMQERFDGRFYWNYFARPGLADAEFARDPRTALRTLLHSASGDAPGAGRPDQALVSDPARGWLADMTDPGELPEWLTEEDLDELTAGYARGFTGALNWYRNLDRNWELTAPWHGAVVTTPALYVYGDRDLVATFPGTPELIAQLPTLLPGLRRPPVMLPGCGHWTQQERPREVNAALVEFLTELRG

**EH25**

MTGPATSTPHVSESNPVRDLPLRHLAGFTHRWVDADGVRLHAVEGGRPNGPTVVLLAGFPQTWWAWRKVMPDLAHRCHVIAIDLPGQGHSARPERSYDTHTVAAHVHTAVQALGVSTYSLIAHDIGAWVAFSLALTFENHLRGVALLDAGIPGITLPEAIPTVPDRAWKTWHFAFHLVPDLPETLLAGREREYVGWFLKMKALSPDTFDDAEVDHYAAAVAADGGLRASLAYYRDAAESARKNREALKGRHLTVPVLGVSSSHGSVPDMAAAISPWADNTTGTVVPDAGHFIPDEQPEAVAAAIADFIADHD

**EH26**

MSDNRIRKLRPVPDAETRMMFRTIHGYRRAFRMAGEGPALLLIHGIGDNSSTWTEIIPHLAKNYTVIAPDLLGHGRSDKPRADYSVAAYANGMRDLLSTLGIDKVTVVGHSLGGGVAMQFSYQFPQMVDRLVLVSAGGVTKDVHPLLRLASVPVVNEALKLLRIPGAMPTVRLVGNVLSQLNGTRLRPGAMLHDTPDLVRVLAELYDPTAYEAYLRTLRAVVDWRGQVVTMLDRCYLTENLPVQLIWGDQDAVIPVSHAHLAHAAMPGSHLEIFRGAGHFPFRDDPMRFLRTIEKFLSGTHPLQFDEAKWRQMLVTGVGESTITGSASTRMAVLDAMGSDERSAT

**EH27**

MRREAPGDARTSGPNGAPDNTFPALGEVRHRYVRLPGLRVHVAEAGHGEPVVLLHGFPQHWWEWRGVVPGLAEHYRVICPDLRGAGWTDAPPTGYTRAQLLADVEALLDALELDRVCLIAHDWGALLGYELCLSAPPRVRKQISLGVPHPFIRFDPRLLTTIARHGWFQPVIAAPFLGPFLLGRGRQRLTDHLLRGFTTDRNTWSERDVELFAGRLREPARAHAASALYRCFIMRQAARIMTGAYRHTRLATPTRVLVGAEDAIVRKEFLGGFEGHTDDFGVEFVDGASHFLVDERPDVVLERALAFFAAA

**EH28**

MAYELDPRFSHHMIDLDEVRLHYVRGGDGPPLVLLHGWGSTWYMWRKVISQLADHFTVIVPDLRGLGDSGVPEKPLTGYDKKTVAQDIHQLVEKLELGPVHVAGHDHGAAVAYAYAATHRDEVRSLVFCEMALKGVAGDKGIEYFMDQRDELRLWHLSFHAASHVAEMLISGKEREYLRWFYRMQMYNLDGITDEDIDVYARSYSGPGGLRLELYRAFYQDGIDNAELSKTKLTIPVLALGGANSIRDLCVSSMEAVADNVRGVVVPNCGHWLPEERPAEFLESVQSFLKEVPA

**EH29**

MSDNATPSTRFGTATPPYAVPRRSKTSIQKHDLKHGLVPVAEGVRLHYVVAGEGEPVLLIPGWPQSWYAWRFIIPLLVEAGRRVYAIDPRGFGDSDMPSEGYGLDNVAEDVHVFIEQLGLAGPDGLDIVSHDTGSWIAHAHAAAYPEDVRTLVLSDAHIPWVSPLPERGYPDDSLNARQWHFYFNRVEGLSEALIHGREREFLSWFFGPSKLARTWVIDAEAFEEYLRVFSKPGAVRAGLNYYREVFSPRGRAASEARKQKRLDMPILTLGGSYADADNLFHTMRQFSTDVRDRVFEGIGHHLPEECPEEMAAEIIDFWAAPPPVRLSGCSSNQNGARARRA

**EH30**

MKPVEPQHHMVWADDIRFHVVEAGEGPTIVLVAGYPQSVYAWRRVIPLLAQNYHVIALDLPGQGDSDKPLGGYDTKTTSARVRALMSAMNQERYLFVGHDIGSWVGYAYAHQFADDLRGLVLLDGNIPGVTLQPTLTLGPDNWRNWHFLFNPIADLPEALLAGRERILIEWFFSRKTANWRATFSREDIDEYERVYQAPGGTRGMLGYYRAVLEDIEQNSSLMTTKIAVPVLALGGEVGSAPDLFERMKPLGTDVRGGLIPRSGHYIPEEEPEALVREISSFAYTLPQ

**EH31**

MNPSRMRRLRPVPNTEPQLTFRTIHGYRRAFRMTGNGPALLLLHGIGDNSTTWTEIIPHLAENYTVIAPDLLGHGRSDKPRADYSVAAYANGIRDLLSTLGIDHATVIGHSLGGGIAMQFAYQFPHMVDRLILVSAGGITKDVHPLLRLAATPILNEALKLLRLPGAVPAVRWVGTVLTRLHGTPLHPGAALHDTPDLVRILTELPDPTAYEAYLRTLRAVVDWRGQTVTMLDRCYLTASLPVQLIWGDRDTVIPVSHAHTAHTAMPGSRLDIFPDAGHFPFRDDPLRFVHTVEKFLSDTRPSPFDEARWRQLLNTGTVQAALTPDASTRATVLDAVGPAESKAT

**EH32**

MAPPDPSTVRFDGPWIHRDIHANGIRFHTVEVGASAPDAPLVILLHGFADFWWSWRHQLTALSEQGYRAVAVDLRGYGDSDKPPRGYDGWTLAGDIAGLIRAMGYGEATLVGHADGGLVCWATAVLHARLVRSIALVSSPHPLALKQAVLHDRYQRKALLPSFVSCQVPWRPERRLTRDNGSEVERLIRERSGPGWTENPEFDVVVSRMRSAIQIPGTAHCTLEYQRWAFRSQFRPDGSRFMASMDQTLRIPVLHLHGDLDPYVLADTVRRSHRFAPTQQMQTVPGVGHYAHWEAPERVNAALLDLVAPRTA

**EH33**

MNPSRMRRLRPVPDAEPQLMFRTIHGYRRAFRMTGEGPALLLLHGIGDNSSTWTEIIPHLAEKYTVIAPDLLGHGRSDKPRADYSVAAYANGMRDLLSTLGIDHATVIGHSLGGGIAMQFAYQFPQMVDRLILVSAGGITTDVHPLLRLAATPILNEALKLLRLPGAVPAVRWAGTLLTRLHGTALHPGAALHDTPDLVRILTELPDPTAYEAYLRTLRAVVDWRGQTVTMLDRCYLTASLPVQLIWGDHDTVIPVSHAHTAHAAMPDSRLDIFPGAGHFPFLDDPMRFLHTVEKFLSDTRPSPFDEARWRQRLNIGTVETVLTPDVSTRAAVLDAVGSAERNAT

Supplementary References

[1] Z. Wang, Y.T. Cui, Z.B. Xu, J. Qu, Hot water-promoted ring-opening of epoxides and aziridines by water and other nucleopliles, J. Org. Chem. 73 (2008) 2270–2274. doi:10.1021/jo702401t.

[2] S. Gao, W. Tang, M. Zhang, C. Wang, J. Xiao, Ru-MACHO-Catalyzed Highly Chemoselective Hydrogenation of α-Keto Esters to 1,2-Diols or α-Hydroxy Esters, Synlett. 27 (2016) 1748–1752. doi:10.1055/s-0035-1561971.
